# Immune-related gene signatures of thin endometrium: a transcriptomic and single-cell study

**DOI:** 10.3389/fendo.2025.1626451

**Published:** 2025-10-03

**Authors:** Yifei Niu, Aiwen Le

**Affiliations:** 1 Department of Gynecology, Shenzhen Nanshan People’s Hospital, Affiliated Nanshan Hospital of Shenzhen University, Shenzhen, Guangdong, China; 2 Youth Innovation Team of Medical Bioinformatics, Shenzhen University Health Science Center, Shenzhen, China

**Keywords:** thin endometrium, RNA sequencing, single-cell RNA-seq, immune dysregulation, endometrial receptivity

## Abstract

**Background:**

Thin endometrium (TE) is associated with impaired endometrial receptivity and reduced rates of successful pregnancy. However, the immune-related transcriptomic alterations underlying TE remain poorly understood. This study aimed to identify differentially expressed genes (DEGs) and immune signatures associated with TE using integrated transcriptomic approaches.

**Results:**

Bulk RNA sequencing of endometrial tissues from TE patients and healthy controls revealed 57 DEGs. Gene Ontology enrichment analysis revealed the involvement of immune activation processes including leukocyte degranulation and natural killer (NK) cell-mediated cytotoxicity. Integration with publicly available single-cell RNA-seq data confirmed increased immune cell infiltration and altered gene expression in stromal and epithelial cell populations. Notably, significant upregulation of CORO1A, GNLY, and GZMA was observed in both datasets and validated using quantitative PCR. These genes are functionally related to cytotoxic immune responses. Canonical senescence markers were not detected, suggesting that immune dysregulation may play a more prominent role than senescence in TE pathogenesis.

**Conclusions:**

This study provides transcriptomic evidence that TE is associated with immune-related alterations, particularly those involving cytotoxic gene activation. The identified genes may serve as potential biomarkers or therapeutic targets for improving endometrial receptivity. These findings offer new insights into the molecular mechanisms of TE and lay the groundwork for future functional investigations.

**Clinical Trial Registration:**

Institutional Review Board Statement: This study was approved by the Ethics Committee of Shenzhen Nanshan Hospital (formerly known as Union Shenzhen Hospital of Huazhong University of Science and Technology at the time of ethical approval), under ethics approval number 072652 (approval date: 26 July 2019). The study was also registered with the China Clinical Trial Registration Center under registration number ChiCTR2000038068. The study was conducted in accordance with the Declaration of Helsinki.

## 1 Introduction

Thin endometrium (TE), typically defined as an endometrial thickness ≤7 mm during the proliferative phase, is a clinically significant condition associated with poor outcomes during embryo implantation, an increased risk of miscarriage, and reduced fertility (1). TE is particularly challenging in the context of assisted reproductive technology (ART), where optimal endometrial receptivity is essential for successful pregnancy (2). Although several clinical factors, such as hormonal insufficiency, inflammation, and vascular dysfunction, have been implicated in TE, the molecular mechanisms underlying its development remain largely unknown (3).

Current treatments, including estrogen supplementation and intrauterine therapies, have shown limited efficacy in reversing TE or improving implantation success in many patients. As a result, there is growing interest in uncovering the gene expression signatures and signaling pathways associated with TE to inform novel therapeutic approaches (4).

High-throughput transcriptomic technologies offer valuable tools for identifying genes and pathways associated with endometrial dysfunction (5). Bulk RNA sequencing (RNA-seq) allows the identification of differentially expressed genes (DEGs) in affected tissues, whereas single-cell RNA sequencing (scRNA-seq) enables the precise mapping of gene expression across diverse endometrial cell types (6). Integration of these datasets provides a more comprehensive view of the cellular heterogeneity and transcriptional alterations present in TE.

In this study, we conducted bulk RNA-seq on endometrial tissues from TE patients and healthy controls to identify DEGs associated with TE (7). Gene Ontology (GO) analysis was performed to assess enriched biological processes, particularly those related to immune regulation and tissue remodeling (8). We further integrated publicly available scRNA-seq data to characterize expression patterns at the single-cell level. The expression of selected genes, including CORO1A, GNLY, and GZMA, was validated using qPCR.

Our findings highlight immune dysregulation and altered gene expression profiles as potential contributors to TE pathogenesis (9). These results provide new insight into the transcriptional landscape of TE and may lead to future functional studies and therapeutic development (10).

These findings enhance our understanding of the molecular mechanisms linking cellular senescence to TE and may reveal potential therapeutic targets to improve endometrial health and fertility. Although structural and hormonal abnormalities have long been implicated in TE, the role of immune dysregulation remains largely underexplored. Our study is among the first to systematically dissect immune-related transcriptomic features of TE using both bulk and single-cell RNA-seq data, highlighting a novel perspective on its pathophysiology.

## 2 Materials and methods

### 2.1 Study subjects and sample collection

Endometrial tissues were collected from three patients diagnosed with thin endometrium (TE) and three control individuals with normal endometrial thickness at Union Shenzhen Hospital of Huazhong University of Science and Technology (now officially renamed Shenzhen Nanshan Hospital) between August 2022 and January 2025.

Participants were eligible if they were under 35 years of age; had regular ovulatory menstrual cycles (26–30 days); were nonsmokers; and had no known metabolic, coagulation, immune, cardiovascular, hepatic, renal, or reproductive tract abnormalities. TE was defined as a maximal endometrial thickness <7 mm. Control participants had an endometrial thickness ≥8 mm and a history of at least one spontaneous full-term pregnancy.

The exclusion criteria included endocrine disorders (e.g., PCOS, thyroid dysfunction, hyperprolactinemia, and diabetes), recent endometrial-impacting treatments (e.g., hormone replacement therapy and chemotherapy), structural uterine abnormalities (e.g., fibroids, adenomyosis, and congenital anomalies), active infections, immunologic diseases, or current pregnancy/lactation.

All participants provided written informed consent prior to enrollment. The study protocol was approved by the Ethics Committee of Shenzhen Nanshan Hospital (Approval No. 072652). Further details of the inclusion and exclusion criteria are available in Supporting Information Data S1. The collected tissues were snap-frozen in liquid nitrogen and stored at −80 °C until use (**Table 1**).

**Table 1:** Patient sample data.

Order number	Age	Pregnancy and birth history	Endometrial thickness (mm)	Menstrual regularity	Menstrual volume			Algomenorrhea	Basic disease	
					Less	Secondary	More		Have	Not have
1	35	G1P1	8	regular	-	yes	-	none	-	not have
2	34	G2P1	8	regular	-	yes	-	none	-	not have
3	33	G6P3	10	regular	-	-	yes	none	-	not have
4	28	G2P0	6	regular	yes	-	-	none	-	not have
5	32	G1P0	5.1	regular	yes	-	-	none	-	not have
6	33	G1P0	4	regular	yes	-	-	none	-	not have

Basic diseases refer to chronic conditions such as hypertension and diabetes.

### 2.2 Data acquisition

We obtained publicly available scRNA-seq data from the National Center for Biotechnology Information (NCBI) Sequence Read Archive (SRA) database (accession number PRJNA730360). We compared these data with our own gene expression data from TE patient samples obtained using RNA-seq to explore the role of cellular senescence-related genes in intimal cell senescence, especially CORO1A expression patterns. The detailed results are presented in **Table 2**.

**Table 2:** Dataset.

ScRNA	Number of cells	Sample book		
		Name	Endometrial thickness	Age
SRR14561662	7679	CT-1	9.4	29
SRR14561663	9826	CT-2	8.9	31
SRR14561664	11959	CT-3	11.8	27
SRR14561665	9998	TE-1	4.1	28
SRR14561666	7137	TE-2	6.2	30
SRR14561667	9827	TE-3	5.5	31

### 2.3 Public single-cell RNA-seq data

**Figure 1:**
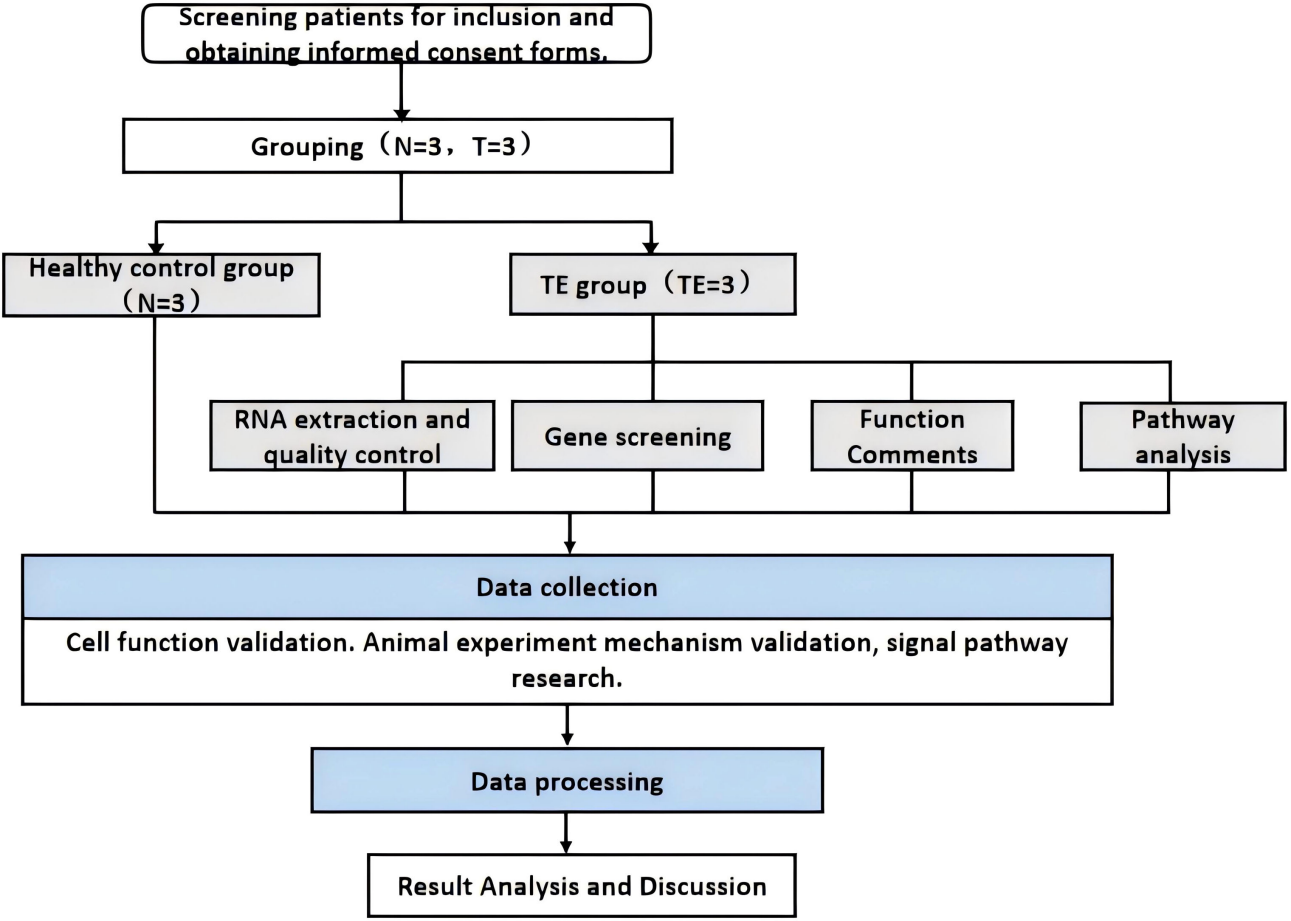
Technical flow chart.

Publicly available scRNA-seq data (accession number: PRJNA730360) were obtained from the NCBI Sequence Read Archive (SRA) database. This dataset contains single-cell transcriptomic profiles of endometrial tissues, including stromal, epithelial, and immune cell types. These data were analyzed and compared with our RNA-seq data to assess the shared expression patterns of genes implicated in TE. This study provides new insights into the transcriptional profile of TE tissues at the molecular level as well as at the cellular level. See **Figure 1** for more details.

### 2.4 Extraction of total RNA from the sequenced samples

Endometrial tissue samples stored at -80°C were quickly transferred to liquid nitrogen, ground into powder, and lysed with 500 μL of RNA-Easy solution. After 200 μL of RNase-free ddH_2_O was added, the mixture was incubated at room temperature, centrifuged to separate the RNA-containing supernatant, and treated with 500 μL of precooled isopropyl alcohol. The RNA was pelleted by centrifugation, washed with 75% ethanol, and dried. It was then dissolved in 40 μL of DEPC-treated water and incubated at 60°C for 10 minutes to obtain total RNA, which was stored at -85-65°C for long-term use or -30-15°C for short-term storage (**Table 3**).

**Table 3:** Instruments and equipment.

Instrument name	Vendor	Country
High-speed centrifuge	American Thermo, Inc	USA
Muller	Shanghai net letter	China
10 µl pipette	Eppendorf	Germany
1 ml pipette	Eppendorf	Germany
Vortex mixer	Dalong Xingchuang Experimental Instrument Company, MX-S	China
-80°C refrigerator	China Meiling Company	China

### 2.5 Library construction and sequencing

Total RNA was extracted from endometrial tissues using RNA-easy isolation reagent (Vazyme) following the manufacturer’s protocol. Ribosomal RNA (rRNA) was removed from the total RNA to obtain mRNA, which was then randomly fragmented in NEB fragmentation buffer using divalent cations. Strand-specific libraries were subsequently constructed from the fragmented mRNA. The concentration and purity of the RNA were assessed using a NanoDrop spectrophotometer. The resulting libraries were diluted to 1.5 ng/μL, and their size distribution was evaluated using an Agilent 2100 Bioanalyzer. Quantitative reverse transcription–PCR (qRT–PCR) was employed to determine the effective concentration of each library, ensuring concentrations above 2 nM to meet the requirements for high-throughput sequencing. Following quality control, the sequencing libraries were pooled and subjected to high-throughput sequencing on the BGISEQ platform, with approximately 6 Gb of data generated per sample. The detailed sequencing metrics are presented in **Table 4**.

**Table 4:** Reagents and consumables.

Reagent	Vendor
RNA-easy isolation kit	Vazyme
Chloroform	In-house
Isopropanol	In-house
DEPC-treated water	Vazyme
Absolute ethyl alcohol	Vazyme

### 2.6 RNA extraction and sequencing

Total RNA was extracted from frozen endometrial tissue using the RNA-easy isolation reagent (Vazyme). RNA integrity was assessed using an Agilent Bioanalyzer. Libraries were constructed using strand-specific methods, and high-throughput sequencing was performed using the BGISEQ platform, generating 6G of data per sample.

### 2.7 RNA-seq data analysis

Raw reads were quality controlled using FastQC, Trim Galore, and Cutadapt. Cleaned reads were aligned to the reference genome using STAR, and gene-level quantification was performed using StringTie and RSEM. The expression levels were normalized using FPKM and TPM metrics.

### 2.8 Differential expression and enrichment analyses

Differentially expressed genes (DEGs) were identified using the DESeq2 package in R. DEGs were defined as those with an adjusted p value (FDR) < 0.05 and a fold change > 1.5. Gene Ontology (GO) enrichment analysis was performed using the clusterProfiler package, with a focus on biological process categories. Visualization was performed using ggplot2. KEGG analysis was not conducted in this study.

### 2.9 scRNA-seq data analysis

scRNA-seq data preprocessing, normalization, dimensionality reduction, and clustering were performed using the Seurat package in R. Quality control excluded cells with low gene counts or high mitochondrial content. Differential expression analysis among cell clusters was conducted using the FindMarkers function, and shared DEGs between bulk RNA-seq and scRNA-seq data were identified using intersect or inner_join functions. Differential expression analysis using the FindMarkers function identified DEGs between clusters. Finally, visualization methods such as t-SNE/UMAP were used to map high-dimensional data, and a heatmap was created to show DEG expression patterns across cell populations, facilitating the identification of gene expression differences (**Table 5**).

**Table 5:** Specific names of the 11 cell clusters identified in the dimensionality reduction and cluster analysis.

Digital label	Cell type
0	B cells
1	Stromal cells
2	Epithelial cells
3	Stromal cells
4	Epithelial cells II
5	Lymphocytes
6	Monocytes
7	Stromal cells
8	Endothelial
9	Ciliated epithelial cells
10	Stromal cells

### 2.10 construction of the gene–biological process network

A gene–biological process network was constructed to visualize the associations between selected DEGs and enriched biological processes related to immune regulation and tissue remodeling. Nodes represent genes and biological processes; edges indicate regulatory relationships.

### 2.11 qPCR validation

Total RNA was reverse-transcribed into cDNA using HiScript III RT SuperMix (Vazyme). qPCR was performed using SYBR Green PCR Master Mix. β-Actin was used as the reference gene. Relative expression levels were calculated using the ΔΔCt method. Primer specificity was confirmed by melting curve analysis. The experiments were conducted in triplicate. Statistical analysis was performed using GraphPad Prism, and significance was determined using t tests (p < 0.05).

### 2.12 Statistical analysis

All analyses were conducted in R (version 4.2.2). Statistical significance was defined as p < 0.05 unless otherwise stated.

## 3 Results

### 3.1 Transcriptome sequencing and gene expression profiling

#### 3.1.1 Identification of differentially expressed genes

**Figure 2:**
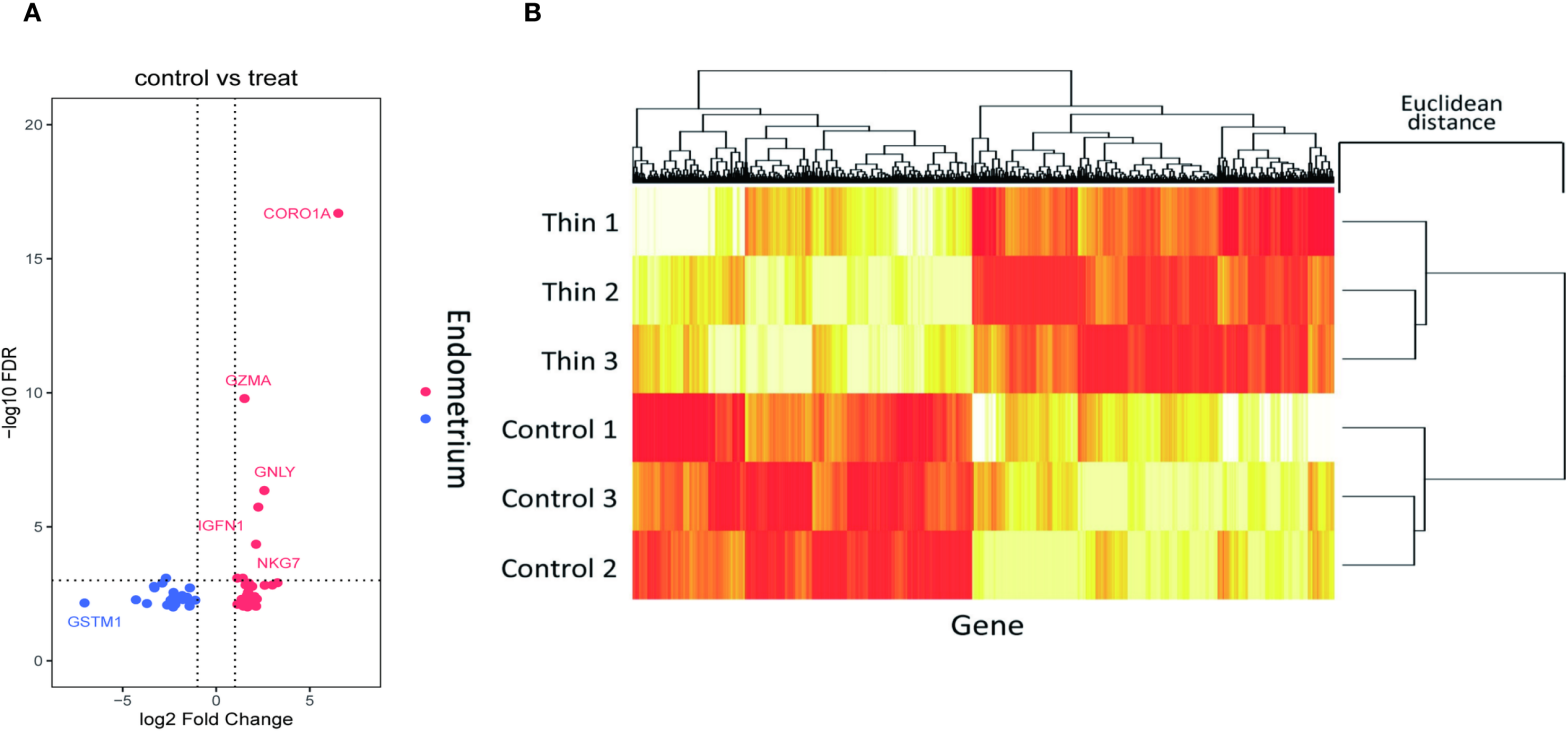
Screening of DEGs in the thin and normal endometrial groups using RNA-seq. **(A)** Volcano plot showing 33 upregulated genes and 24 downregulated genes; red represents upregulation, and blue represents downregulation. **(B)** Cluster heatmap showing the expression of the upregulated and downregulated genes in the two samples. The x axis represents the genes, and the y axis represents the samples. Red represents high expression, and yellow represents low expression.

We analyzed RNA-seq data from six endometrial tissue samples, including three thin endometrium (TE) and three control (CT) samples. DEGs between TE and CT tissues were identified using the Limma package in R, applying a threshold of |log_2_ fold change| > 1 and p < 0.05. A total of 57 significant DEGs were detected, including 33 upregulated and 24 downregulated genes (**Figure 2**).

The upregulated genes were primarily associated with immune-related functions, including cell migration and activation, whereas the downregulated genes were involved in pathways related to cell proliferation and signal transduction. These findings suggest that TE may be characterized by altered immune responses and suppressed cellular growth signaling.

#### 3.1.2 Gene ontology enrichment analysis

**Figure 3:**
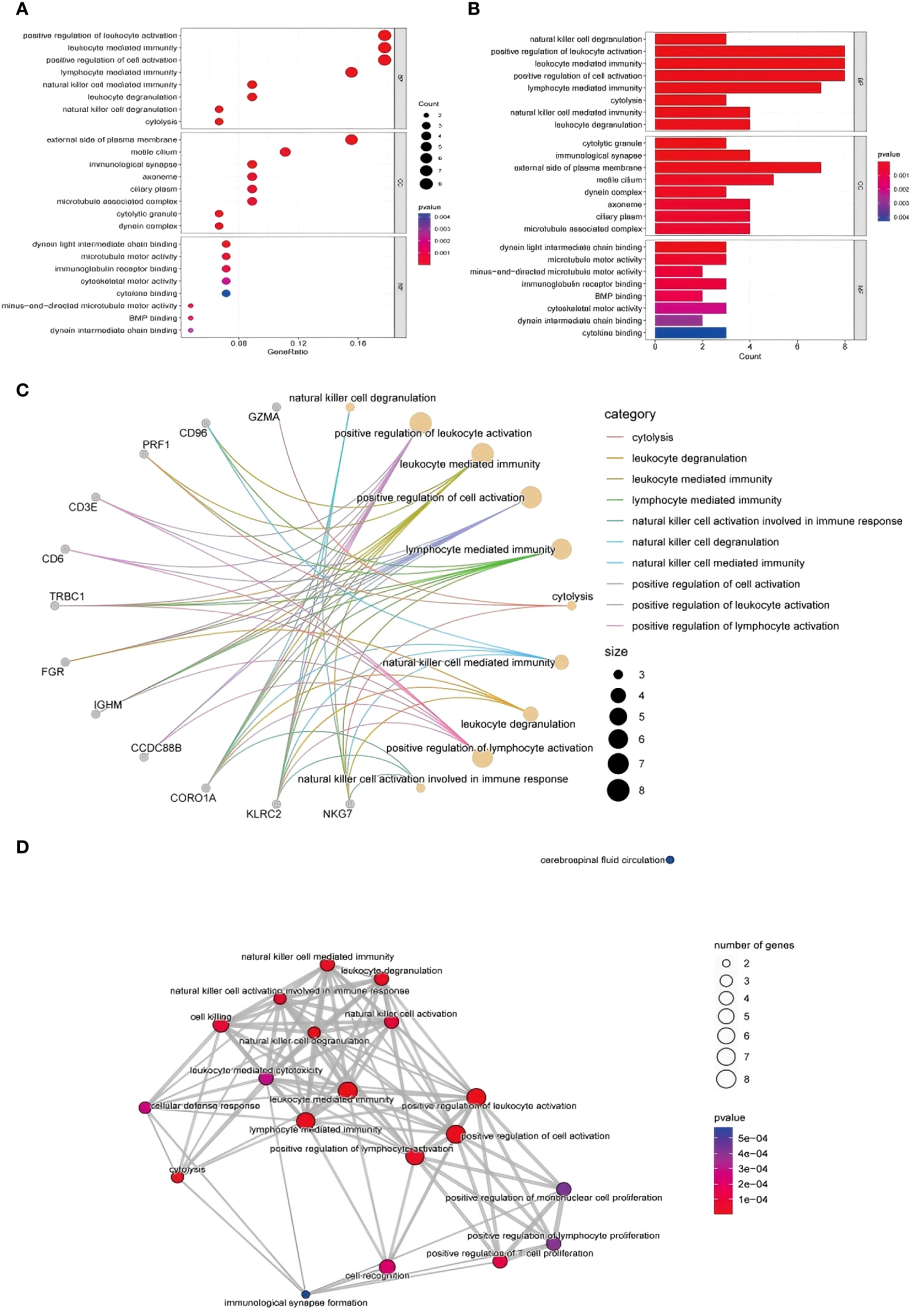
GO functional enrichment analysis of DEGs identified via RNA-seq. **(A, B)** GO analysis of upregulated genes and the biological process, cell component and molecular function results: from top to bottom, biological process, cell component, and molecular function enrichment analysis results: Aba indicates the number of genes enriched to an entry, and the color of the points from red to blue represents the P value change from small to large; **(C)** gene–biological process network diagram; **(D)** enrichment of biological processes related to the immune response.

To elucidate the functional implications of the DEGs, GO enrichment analysis was performed, and the terms were classified into three categories: biological processes (BP), molecular functions (MF), and cellular components (CC). Enrichment analysis of the upregulated genes revealed strong associations with immune responses, inflammatory signaling, oxidative stress, and cell cycle regulation (**Figure 3A** illustrates the relevant findings here). Notably, genes were enriched in processes such as natural killer (NK) cell activation, immune cell-mediated cytotoxicity, and oxidative stress responses.

In terms of molecular function, upregulated genes were involved in ATP binding and DNA repair activities, whereas cellular component enrichment highlighted roles in cytoplasmic and nuclear processes. The downregulated genes were enriched in pathways related to cell proliferation, signal transduction, and transcriptional regulation. These patterns may reflect impaired regenerative capacity and altered immune balance in TE tissues (**Figure 3B** illustrates the relevant findings).

#### 3.1.3 Gene–biological process network analysis

To visualize the relationships between the DEGs and enriched biological processes, we constructed a gene–biological process network (**Figure 3C** illustrates the relevant findings). Genes such as CD96, GZMA, and CORO1A were prominently linked to immune-related processes, including natural killer cell-mediated immunity, leukocyte degranulation, and lymphocyte activation (11).

CORO1A, in particular, was strongly associated with NK cell-mediated responses, suggesting a potential role in immune modulation in the context of TE. Similarly, CD96 was connected to leukocyte degranulation, and GZMA is involved in cytolytic activity. These interactions highlight the relevance of immune cell functions in TE-associated gene expression changes.

#### 3.1.4 Enrichment of immune-related biological processes

Further network analysis revealed enrichment of several biological processes related to immune activation, including NK cell degranulation, lymphocyte-mediated cytotoxicity, and T-cell proliferation (**Figure 3D** illustrates the relevant findings). These findings point to a potential shift in immune dynamics in TE tissue, particularly in NK and T-cell function. Genes such as PRF1, NKG7, and CD3E were significantly involved in cytolytic pathways, suggesting that TE may be characterized by enhanced immune effector activity and altered immune regulation.

In addition, the network included biological processes associated with cell proliferation and activation. Enrichment of pathways such as monocyte proliferation and positive regulation of lymphocyte activation may reflect altered immune cell dynamics and impaired tissue regeneration in TE (12). Cytolysis-related gene expression patterns suggest increased clearance of dysfunctional or senescent cells, potentially linked to tissue remodeling processes in TE.

Together, these results indicate that TE is associated with dysregulated immune signaling and altered cell cycle activity. The identified DEGs and their involvement in immune-related biological processes offer a foundation for understanding TE pathophysiology and identifying potential biomarkers for future studies (13).

### 3.2 Single-cell RNA-seq analysis

#### 3.2.1 Data quality control and differential gene expression

**Figure 4:**
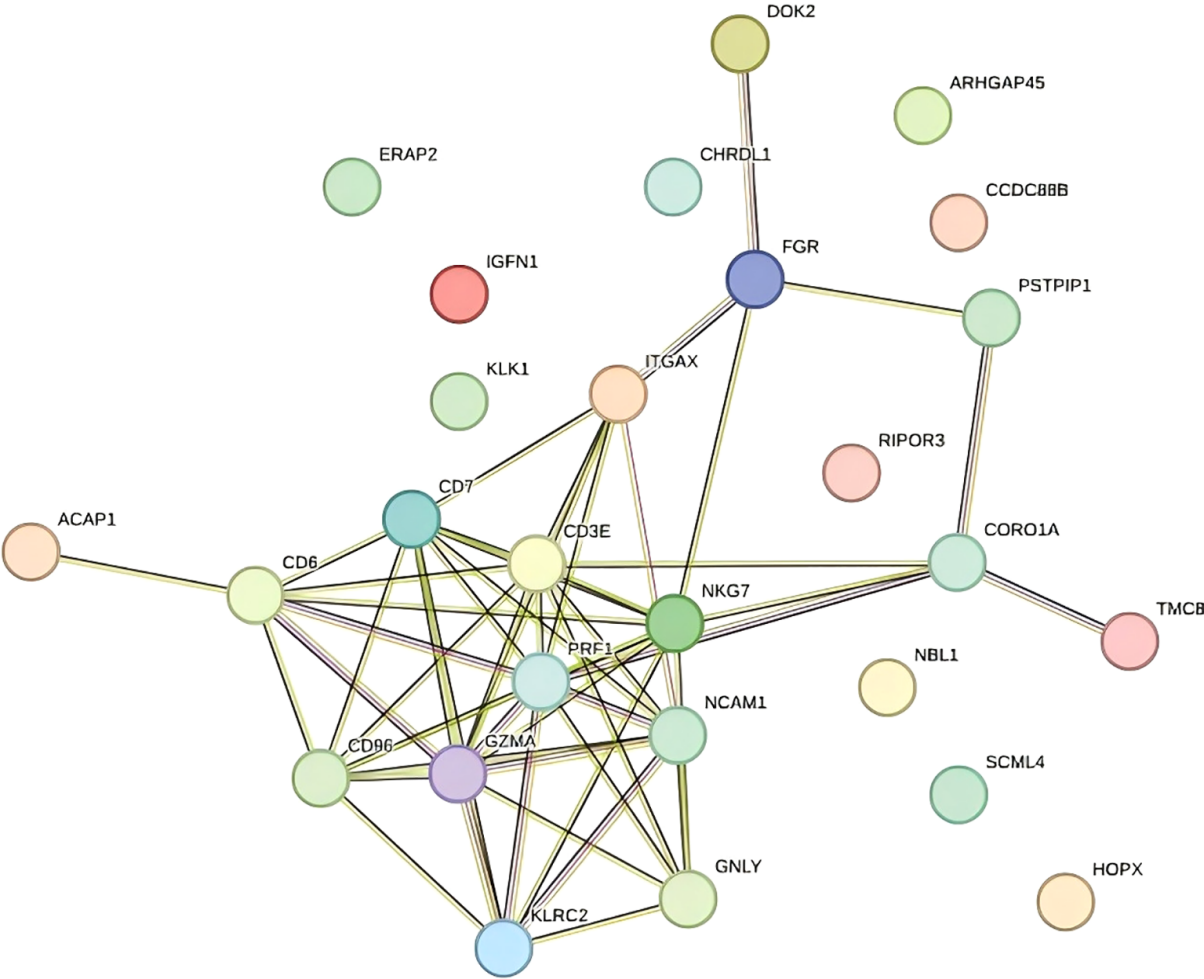
Protein–protein interaction network (PPI).

Analysis Publicly available scRNA-seq data (accession: PRJNA730360) were processed using the Seurat package in R. Quality filtering excluded cells with <500 or >5000 genes or >20% mitochondrial gene content. After normalization and scaling, differential gene expression analysis was performed between TE and CT tissues. Shared DEGs identified in both the bulk and single-cell data included CORO1A, GZMA, and GNLY, which exhibited significantly upregulated expression in TE samples (**Figure 4** illustrates the relevant findings). These genes are involved in immune activation and cytotoxic responses, supporting a role for immune alterations in TE.

#### 3.2.2 Functional differences in cell populations

**Figure 5:**
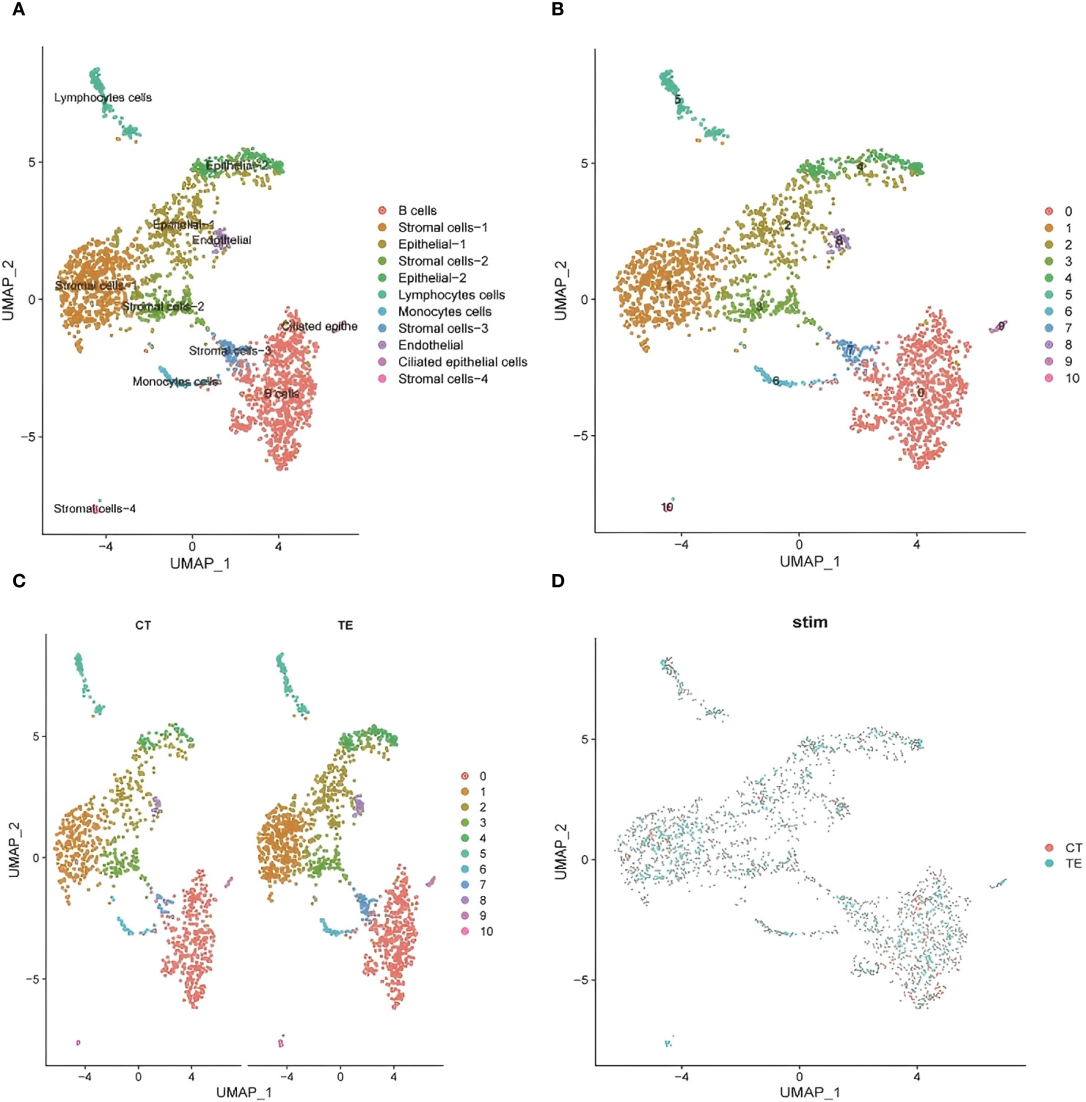
UMAP dimensionality reduction analysis of the single-cell transcriptome data revealed the heterogeneous distribution of cell subpopulations. **(A)** UMAP cluster map of scRNA-seq data (cell type annotation); **(B, C)** cell distribution in CT and TE tissues via UMAP analysis; **(D)** UMAP analysis of the distribution of subpopulations between TE and CT tissues.

UMAP-based annotation identified immune cell subsets including B cells, lymphocytes, monocytes, and stromal cells (**Figure 5A** illustrates the relevant findings). TE tissues were enriched in B cells and monocytes, indicating a proinflammatory microenvironment. In contrast, CT samples showed a more stable immune profile (14). These data suggest that TE is associated with increased immune activation, which is consistent with the transcriptome findings.

#### 3.2.3 Cellular landscape characterization via UMAP

To explore tissue heterogeneity, uniform manifold approximation and projection (UMAP) was used for dimensionality reduction and visualization. A total of 11 distinct cell populations were identified on the basis of transcriptomic clustering (see **Figure 5B**). Comparisons of TE and CT tissue samples revealed differences in cell type proportions. TE samples showed increased immune and stromal cell populations, whereas CT tissues had more evenly distributed epithelial cells (**Figures 5C, D** illustrates the relevant findings). This shift suggests altered immune microenvironments and cellular remodeling in TE.

#### 3.2.4 t-SNE visualization of the cell population structure

**Figure 6:**
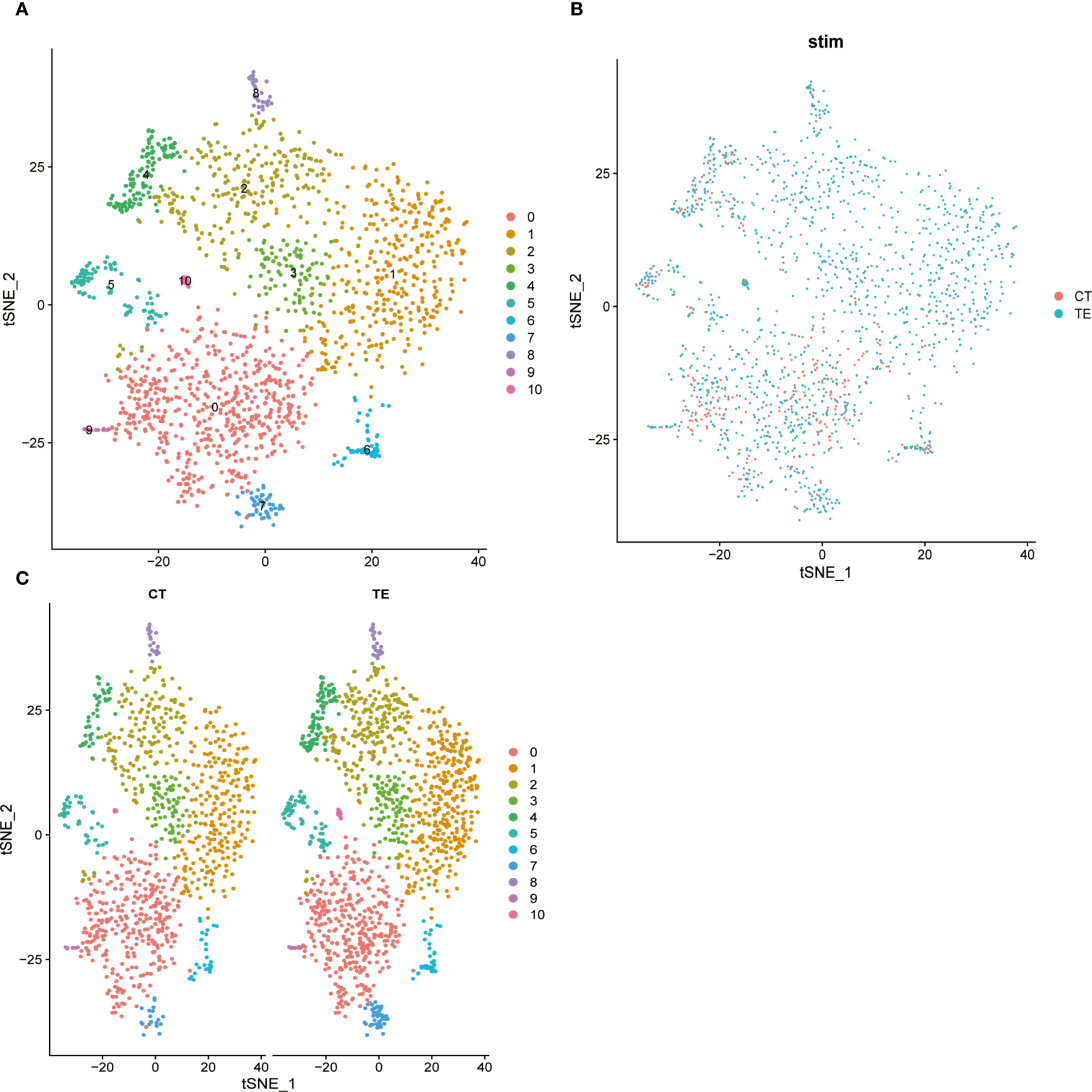
Dimension reduction for all cells using t-SNE and cluster analysis. Different colored dots represent different cell populations, and the apparent separation between populations indicates differences in gene expression between cell populations. **(A)** t-SNE dimensionality analysis of single-cell transcriptome data from TE tissues; **(B)** t-SNE dimensionality analysis of single-cell transcriptome data from endometrial tissues; **(C)** t-SNE shows the distribution of different cell subsets in TE and CT tissues.

To complement the UMAP analysis, t-SNE was used to visualize transcriptomic differences. The cell clusters were clearly separated, reflecting expression-based heterogeneity (**Figures 6A-C** illustrates the relevant findings). TE tissue cells appeared more concentrated in distinct clusters and were enriched in immune-related cell types. CT tissues showed more diffuse clustering, with fewer immune cells. These patterns further support immune dysregulation and structural changes in TE.

#### 3.2.5 Validation of shared DEGs across datasets

**Figure 7:**
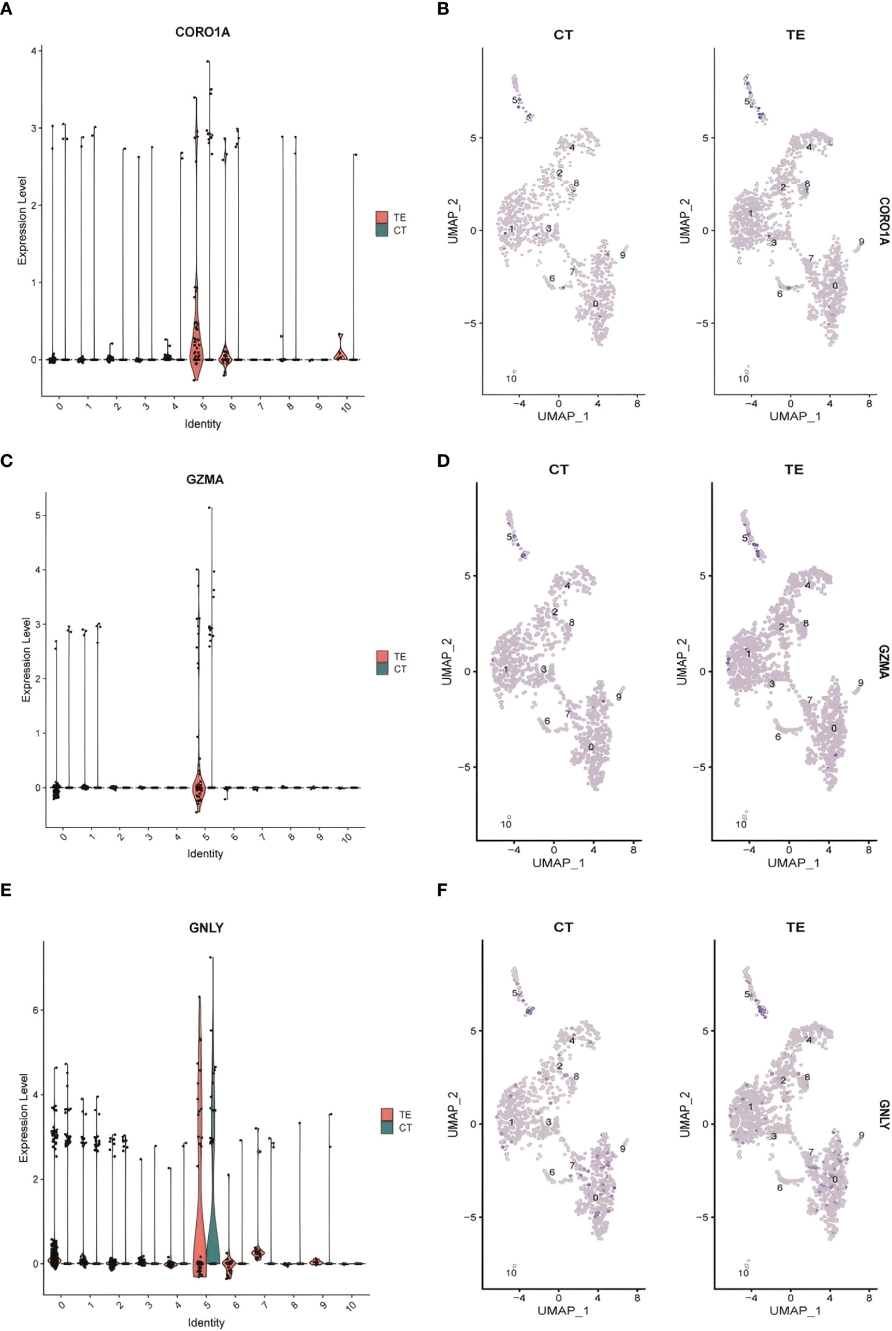
Cluster analysis results for the single-cell data. **(A)** CORO1A expression levels in different cell types; **(B)** differences in CORO1A expression in different cell populations in TE and CT tissues; **(C)** GZMA expression levels in different cell types; **(D)** GZMA expression levels in TE and CT tissues; **(E)** GNLY expression levels in different cell types; **(F)** differential expression of GNLY in different cell populations in TE and CT tissues.

Three DEGs—CORO1A, GNLY, and GZMA—were validated across both the bulk RNA-seq and scRNA-seq datasets. Violin plots and UMAP visualizations revealed elevated expression of these genes in TE tissues, especially within epithelial and immune cell clusters (**Figures 7A-F** illustrates the relevant findings). Although canonical senescence markers (e.g., CDKN2A/p16 and CDKN1A/p21) were not differentially expressed, immune-modulatory genes such as CORO1A, GNLY, and GZMA may play roles in immune responses associated with tissue remodeling.

#### 3.2.6 GO enrichment analysis of scRNA-seq data

**Figure 8:**
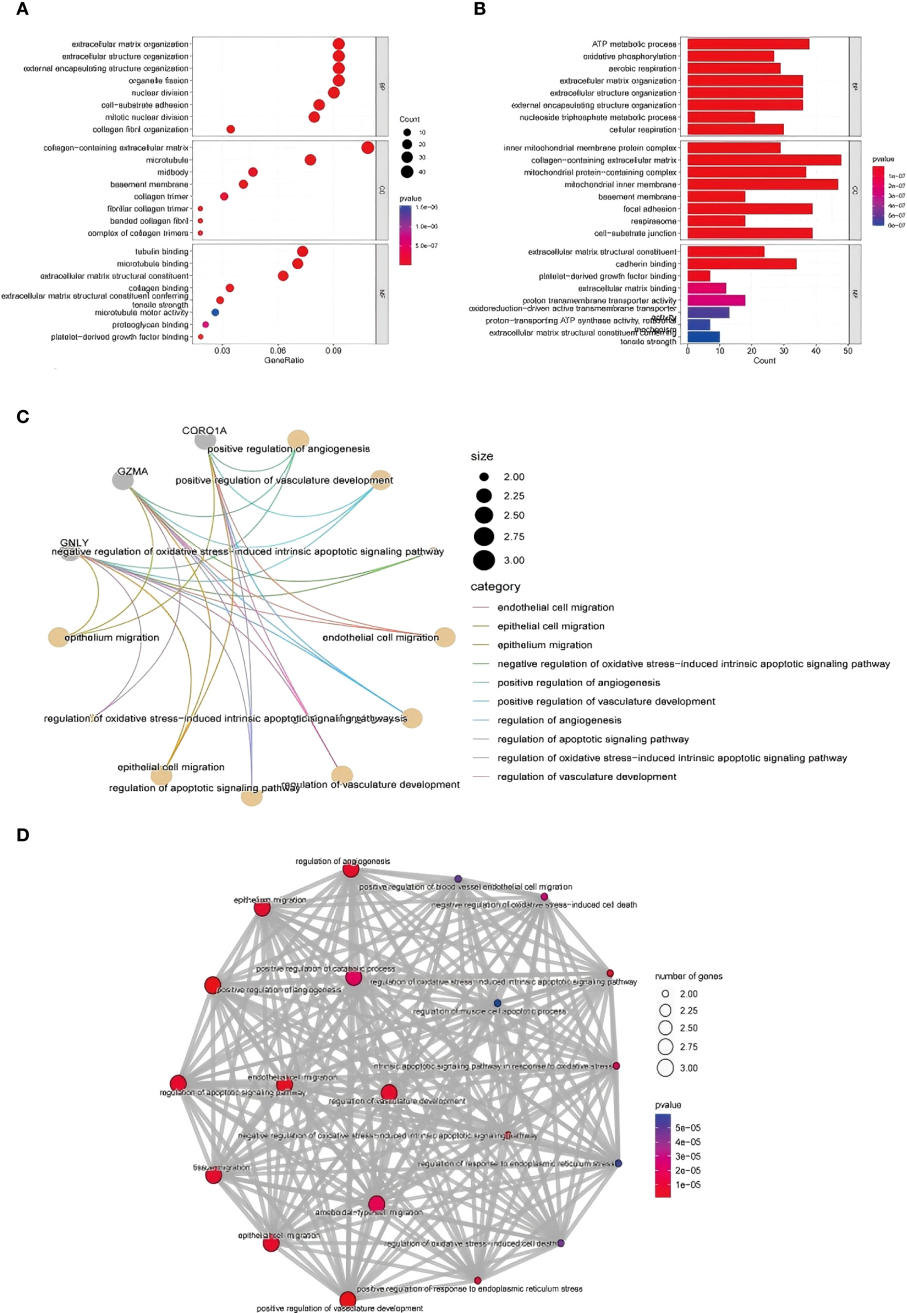
GO functional enrichment analysis of the DEGs identified using scRNA-seq. **(A, B)** GO enrichment analysis results. The enriched biological process, cellular component, and molecular function terms are listed from top to bottom. The abscissa is the number of genes in an entry, and the color from red to blue represents the p value from small to large, respectively. **(C)** shows the interaction of key immune-related genes like CORO1A, GNLY, and GZMA. **(D)** highlights the involvement of vascular development, apoptosis, and ECM-related processes in TE tissues.

GO enrichment analysis of DEGs from the scRNA-seq data revealed significant terms related to extracellular matrix (ECM) organization, cell adhesion, and ATP metabolism (**Figures 8A-C** illustrates the relevant findings). In TE tissues, enrichment of ECM-related terms suggested dynamic but potentially impaired remodeling. The enrichment of genes involved in basement membrane and cell–matrix contact further supported altered tissue structure.

In terms of molecular function, TE-associated genes were enriched in collagen and microtubule binding activities, reflecting cytoskeletal remodeling and possible fibrosis. In contrast, CT tissues were enriched in ECM structure and redox transport, indicating stable metabolic and structural homeostasis. These findings reinforce that TE may involve abnormal immune activity and tissue remodeling at the single-cell level (**Figure 8D**).

Together, these analyses support the conclusion that TE is characterized by increased immune cell infiltration and altered expression of genes related to immune regulation and ECM remodeling. The integration of bulk and scRNA-seq data enhances our understanding of the cellular diversity and molecular pathways implicated in TE pathophysiology.

### 3.3 Validation of differential gene expression by qPCR

#### 3.3.1 Validation of CORO1A, GNLY, and GZMA expression

**Figure 9:**
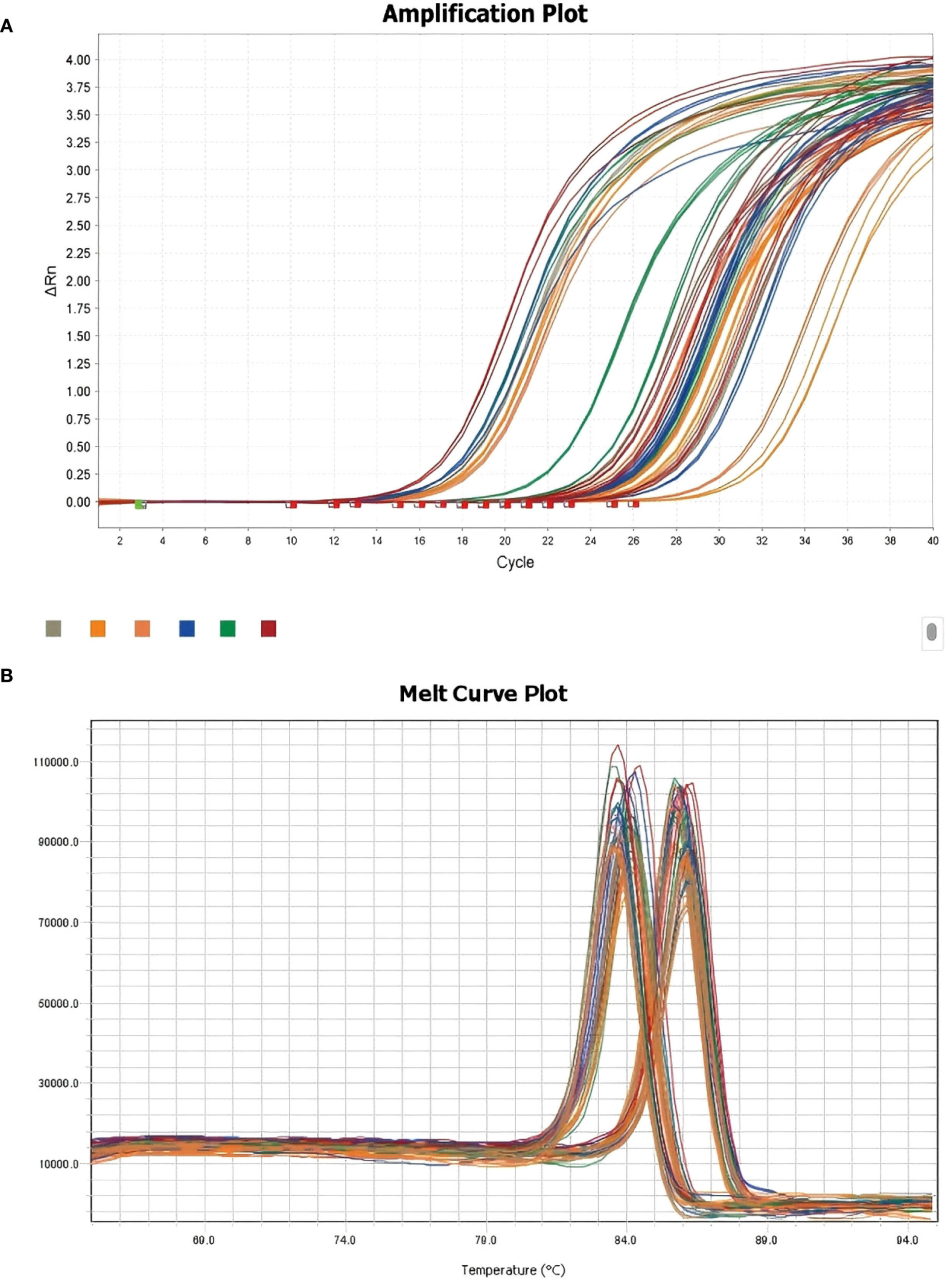
Melting curve plots of the qPCR results. **(A)** qPCR-integrated amplification curve; **(B)** qPCR-integrated melting curve.

To validate the transcriptomic findings, we performed quantitative PCR (qPCR) to measure the expression levels of CORO1A, GNLY, and GZMA in TE and CT tissues. β-actin was used as the internal reference. Amplification curves revealed robust fluorescence signals across all the genes, and consistent Ct values were observed, indicating successful and efficient amplification (**Figure 9A** illustrates the relevant findings).

Melting curve analysis revealed single, distinct peaks for each gene, confirming the specificity of primer design and the absence of nonspecific amplification (**Figure 9B** illustrates the relevant findings). These results validate the technical reliability of the qPCR assays.

#### 3.3.2 Quantitative expression analysis and visualization

**Figure 10:**
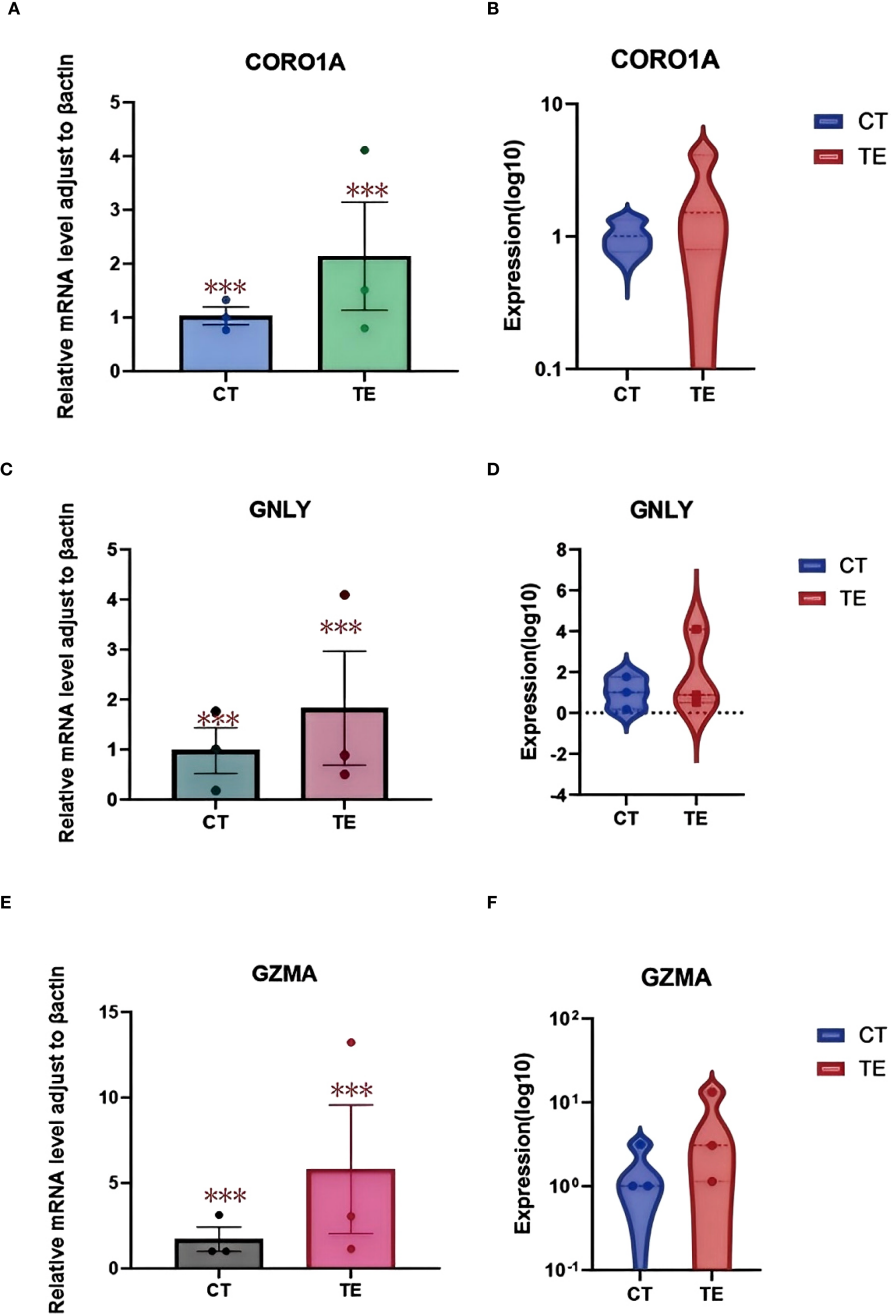
qPCR validation results. **(A)** Boxplot showing the relative mRNA expression levels of the CORO1A gene in TE and CT tissues; **(B)** violin plots showing the log10-transformed expression of the CORO1A gene in TE and CT tissues. **(C)** boxplot showing the relative mRNA expression levels of the GNLY gene in TE and CT tissues; **(D)** violin plots showing the log10 expression of the CORO1A gene in TE and CT tissues; **(E)** boxplot showing the relative mRNA expression levels of the GZMA gene in TE and CT tissues; **(F)** violin plots showing the difference in log10-transformed expression of CORO1A genes in TE and CT tissues. GraphPad Prism was used to perform t tests to compare the differences in gene expression between the TE and CT groups. A p value less than 0.05 indicated a statistically significant difference. The special symbol * * *indicates a p value less than 0.001.

Relative quantification revealed that all three genes were significantly upregulated in TE samples compared with those in CT controls. Specifically, the expression level of CORO1A was approximately 2.5-fold greater in TE tissues (p < 0.01), that of GNLY was approximately 3.1-fold greater (p < 0.01), and that of GZMA was approximately 2.8-fold greater (p < 0.01) (**Figures 10A, C, E** illustrates the relevant findings).

Boxplots and violin plots were used to further visualize expression differences. Violin plots demonstrated tighter expression distributions for CORO1A in the TE group, indicating consistent upregulation, whereas GNLY and GZMA showed greater variability but similarly elevated levels (**Figures 10 B, D, F** illustrates the relevant findings). All differences were statistically significant (p < 0.001).

These qPCR results confirm the differential expression of CORO1A, GNLY, and GZMA identified using RNA-seq and scRNA-seq. Given their known roles in immune regulation and cytotoxic responses, these genes may serve as key contributors to the altered immune landscape in TE tissues. These findings support the hypothesis that immune-related gene expression changes are involved in TE pathophysiology and warrant further investigation.

## 4 Discussion

Thin endometrium (TE) is a significant concern in female reproductive health, particularly because it is associated with clinical issues such as infertility, recurrent miscarriage, and adverse pregnancy outcomes (15). The endometrium plays a crucial role in the menstrual cycle and undergoes complex processes such as hyperplasia, secretion, and shedding, which are essential for successful fertilization and embryo implantation (7). Typically, TE is defined as an endometrial thickness of less than 4 mm, and its presence is often linked to impaired endometrial repair and regeneration capacity (16). Despite the considerable clinical studies focused on the treatment and management of TE, the molecular mechanisms that underlie the development of TE and the regulation of cellular senescence in the context of TE remain poorly understood.

In recent years, the application of high-throughput sequencing technologies has significantly advanced the understanding of gene expression changes in various tissues, including the endometrium (17). These approaches have allowed the identification of key regulatory genes involved in endometrial repair, regeneration, and aging. In particular, high-throughput RNA sequencing was used to investigate gene expression in TE tissue, with a focus on identifying potential molecular regulators (18). These results suggest that genes such as CORO1A, GNLY, and GZMA play important roles in immune response regulation, cell migration, and tissue remodeling in TE. These findings are crucial because they suggest that dysregulated cellular processes, such as altered immune activation and inflammatory responses, may contribute to the development of TE and impair tissue regeneration (19).

The identification of CORO1A as a significantly upregulated gene in TE tissue highlights its potential importance in the pathophysiology of TE (20). CORO1A, which is involved in actin dynamics and immune cell migration, plays a pivotal role in modulating immune responses by supporting the structural integrity and function of cytoskeletal proteins in T and natural killer (NK) cells (21). The upregulation of CORO1A expression may increase immune cell infiltration, potentially altering the local immune environment of the endometrium and disrupting its regenerative capacity. In parallel, the upregulation of GNLY and GZMA, key mediators of NK and cytotoxic T-cell activity, suggests an enhanced cytolytic immune profile (1). These genes contribute to immune-mediated cell clearance mechanisms, and their increased expression may reflect attempts to eliminate damaged or dysfunctional cells within the endometrium (22). However, persistent activation of such responses may also impair tissue recovery and exacerbate structural deficits in TE.

Notably, the consistent upregulation of these genes was validated across bulk RNA-seq, single-cell RNA-seq, and qPCR experiments, enhancing the robustness of our findings. Previous studies have associated CORO1A, GNLY, and GZMA with immune surveillance and cytotoxic responses, particularly in inflammatory and fibrotic diseases. Although our data do not establish direct causality or functional effects, they highlight transcriptional signatures that may accompany or influence TE pathology (23). These genes may reflect immune activation states in TE and represent preliminary leads for future biomarker discovery or therapeutic investigations, pending functional validation. It remains unclear whether immune activation precedes and contributes to TE development or arises because of tissue thinning; this question warrants further longitudinal and mechanistic investigation.

The molecular mechanisms underlying TE are multifaceted and likely involve complex interactions between immune cells, the extracellular matrix, and endometrial epithelial or stromal cells. The findings of this study contribute to the growing body of evidence suggesting that immune dysregulation and aberrant tissue remodeling are key features of TE (24). The upregulation of CORO1A, GNLY, and GZMA may thus reflect immune-related perturbations rather than direct drivers of TE, but they offer promising leads for further mechanistic studies.

This study has several limitations. First, transcriptomic data offer insight into gene expression but do not confirm protein-level function. Future studies should include Western blotting or immunohistochemistry to confirm the translation and localization of the identified genes. Second, the relatively small sample size limits the generalizability of these findings (25). Larger cohorts and animal models are necessary to verify these results and determine their biological relevance. Moreover, the functional roles of CORO1A, GNLY, and GZMA in TE should be investigated using *in vitro* assays and perturbation experiments to clarify whether they contribute causally to tissue remodeling or immune dysfunction.

**Figure 11:**
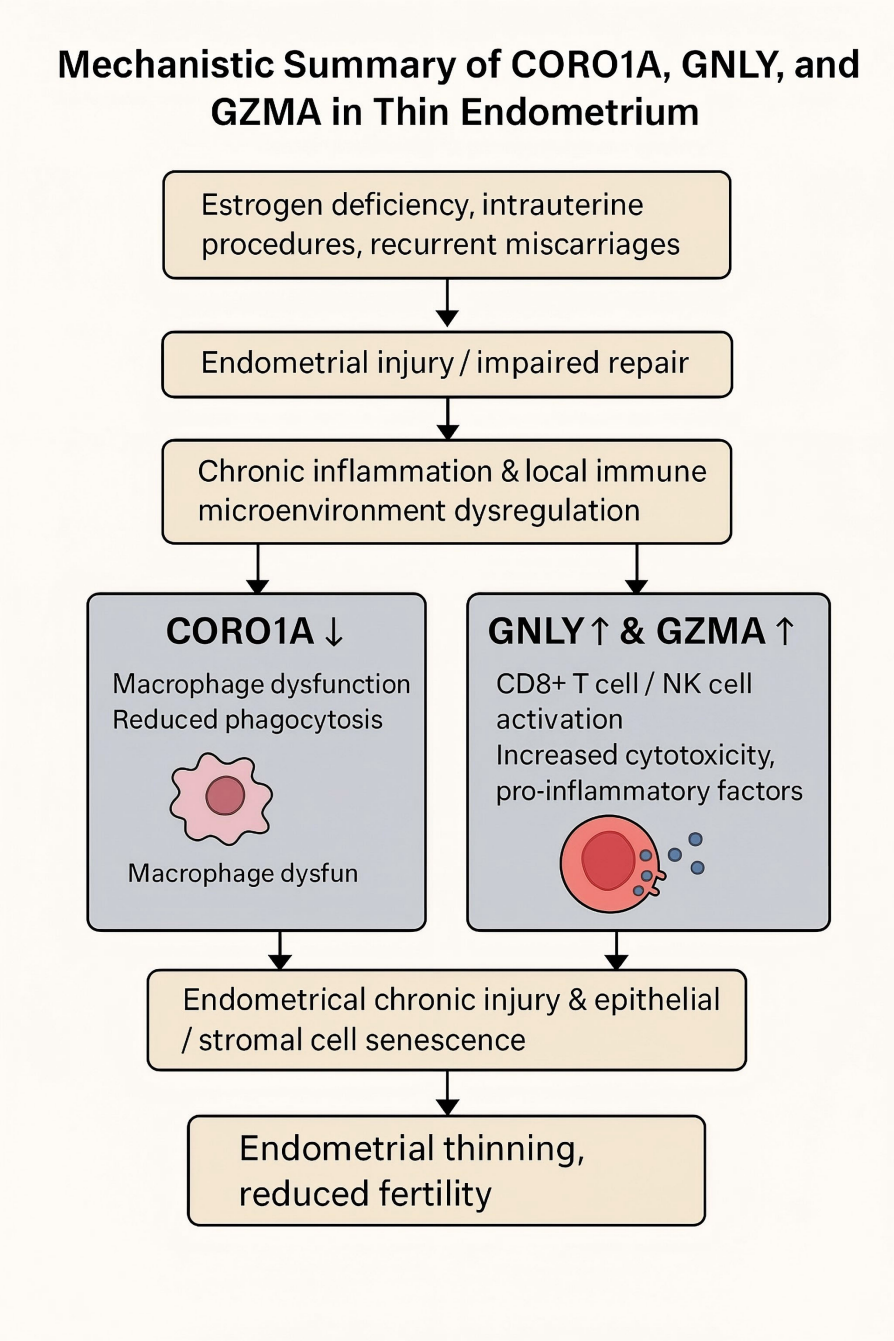
This diagram illustrates the proposed immunological mechanism contributing to a thin endometrium (TE). Decreases in CORO1A expression may lead to macrophage dysfunction and impaired phagocytosis, while upregulation of GNLY and GZMA expression may promote cytotoxicity and proinflammatory signaling via CD8+ T and NK cells. Together, these changes may result in chronic endometrial injury, cellular senescence, and impaired tissue regeneration.

As summarized in **Figure 11**, we propose a hypothetical immunological mechanism linking CORO1A, GNLY, and GZMA to endometrial thinning through macrophage dysfunction, cytotoxic activity, and epithelial/stromal senescence. Finally, future research should explore the signaling pathways modulated by these genes, particularly those governing immune–endometrial cell interactions and local inflammatory responses. Advanced technologies such as spatial transcriptomics or multiplexed immunofluorescence could further elucidate the cellular microenvironment and enhance our understanding of TE pathophysiology. In future studies, functional experiments will be conducted to validate the roles of key immune-related hub genes. For instance, CRISPR/Cas9-mediated knockout of CORO1A in human endometrial stromal cells may help determine its regulatory effect on immune signaling pathways and cell proliferation. Similarly, spatial transcriptomic techniques, such as 10x Genomics Visium or MERFISH, could be employed to localize GNLY- and GZMA-expressing immune cell subsets within the endometrial microenvironment, thereby elucidating their spatial distribution and interactions with stromal and epithelial cells. These strategies contribute to a more in-depth understanding of the immune landscape and functional mechanisms underlying TE.

In conclusion, our study highlights CORO1A, GNLY, and GZMA upregulation in thin endometrial tissue and suggests that these genes may participate in immune-related pathways associated with impaired tissue regeneration (26). These results provide molecular insights that may inform future biomarker discovery and therapeutic targeting to improve endometrial receptivity and fertility outcomes. Our sample size was relatively small because of the strict inclusion/exclusion criteria and the difficulty in obtaining high-quality, cycle-matched endometrial tissue. As such, the findings should be interpreted as exploratory and hypothesis generating. Future studies involving larger and longitudinal cohorts will be needed to validate these transcriptomic signatures and improve generalizability. In future studies, we plan to validate key immune-related hub genes (e.g., CORO1A, GNLY, GZMA) at the protein level using Western blotting and immunohistochemistry, as outlined in **Supplementary Data S2**.

## Data Availability

The original contributions presented in the study are included in the article/**Supplementary Material**, further inquiries can be directed to the corresponding author/s.
