# Supplementary material for: Immune-related gene signatures of thin endometrium: a transcriptomic and single-cell study
Source: Front Endocrinol (Lausanne). 2025 Oct 3;16:1626451. doi: 10.3389/fendo.2025.1626451 (PMC12531038; doi:10.3389/fendo.2025.1626451)
Supplement: Supplementary file 1 [file DataSheet1.docx]

# Supporting Information Data S1

This supplementary file provides detailed inclusion and exclusion criteria, as well as baseline characteristics of the endometrial tissue samples used in this study.

## 1. Inclusion Criteria

- Female, age < 35 years

- Regular ovulatory menstrual cycles (26–30 days)

- Endometrial thickness <7 mm (TE group) or ≥8 mm (Control group)

- No hormonal treatment within 3 months

- At least one full-term pregnancy (Control group only)

## 2. Exclusion Criteria

- Polycystic ovary syndrome (PCOS)

- Thyroid dysfunction, hyperprolactinemia

- Uterine fibroids, adenomyosis, endometrial polyps

- History of chemotherapy, radiotherapy, or immunotherapy

- Active infection or autoimmune disease

- Pregnancy or lactation

## 3. Sample Collection and Handling

Endometrial tissues were obtained during the mid-luteal phase (Days 19–23) via curettage. Tissues were immediately frozen in liquid nitrogen and stored at –80°C. Samples were used for RNA extraction and transcriptomic profiling.

## 4. Clinical Data Summary Table

| Order No. | Age | Gravidity/Parity | Endometrial Thickness (mm) | Menstrual Status | Menstrual Volume | Dysmenorrhea | Basic Diseases |
| --- | --- | --- | --- | --- | --- | --- | --- |
| 1 | 35 | G1P1 | 8 | regular | normal | no | none |
| 2 | 34 | G2P1 | 8 | regular | normal | no | none |
| 3 | 33 | G6P3 | 10 | regular | heavy | no | none |
| 4 | 28 | G2P0 | 6 | regular | light | no | none |
| 5 | 32 | G1P0 | 5.1 | regular | light | no | none |
| 6 | 33 | G1P0 | 4 | regular | light | no | none |
