# Supplementary material for: Immune-related gene signatures of thin endometrium: a transcriptomic and single-cell study
Source: Front Endocrinol (Lausanne). 2025 Oct 3;16:1626451. doi: 10.3389/fendo.2025.1626451 (PMC12531038; doi:10.3389/fendo.2025.1626451)
Supplement: Supplementary file 2 [file DataSheet2.docx]

# Supplementary Data S2: Proposed Experimental Design for Protein-Level Validation

To validate the transcriptomic findings at the protein level, we propose follow-up experiments focusing on key immune-related markers identified in this study, including CORO1A, GNLY, and GZMA.

## Sample Source

Fresh-frozen or paraffin-embedded endometrial tissue samples will be collected from additional patients with thin endometrium (n ≥ 6) and matched controls (n ≥ 6), following the same inclusion/exclusion criteria described in the main study.

## Western Blotting (WB)

Target proteins: CORO1A, GNLY, GZMA

Primary antibodies (tentative):

• anti-CORO1A (1:1000, [Vendor, Cat#])

• anti-GNLY (1:1000, [Vendor, Cat#])

• anti-GZMA (1:1000, [Vendor, Cat#])

Normalization: β-actin or GAPDH

Analysis: Relative band intensity quantified using ImageJ

## Immunohistochemistry (IHC)

Sections: 4μm-thick paraffin sections from TE and control tissues

Staining protocol: Standard DAB detection, hematoxylin counterstain

Quantification: H-score or percentage of positively stained cells

Interpretation: Two independent observers blinded to sample identity

## Statistical Analysis

Differences between TE and control groups will be assessed using unpaired t-tests or Mann–Whitney U tests, depending on data distribution.

This follow-up validation will provide critical protein-level support for the transcriptomic signatures identified and further clarify the immunological mechanisms underlying thin endometrium.
